# Feasibility and cost effectiveness of ambulatory laparoscopic cholecystectomy. A retrospective cohort study

**DOI:** 10.1016/j.amsu.2020.04.036

**Published:** 2020-05-12

**Authors:** Tommaso Maria Manzia, Claudia Quaranta, Vincenzino Filingeri, Luca Toti, Alessandro Anselmo, Laura Tariciotti, Gerardo De Carolis, Roberto Cacciola, Nicola Di Lorenzo, Roberto Sorge, Roberta Angelico, Giovanni Monteleone, Giuseppe Tisone

**Affiliations:** a Department of Surgery, HPB and Transplant Unit, Fondazione PTV, Tor Vergata University of Rome, Italy; b Health Management, Fondazione PTV, Rome, Italy; c Laboratory of Biometry, Department of Systems Medicine, Tor Vergata University of Rome, Italy; d Department of Systems Medicine, Tor Vergata University of Rome, Italy

**Keywords:** Ambulatory, Laparoscopic cholecystectomy, Cost evaluation

## Abstract

Ambulatory surgery is an efficient, safe and widely performed procedure; this study would shows the advantages of the ambulatory laparoscopic cholecystectomy procedure from the point of view of patients and the Hospital/National Health System. *Materials and Methods*: Single-center retrospective cohort study including 288 patients who underwent laparoscopic-cholecystectomy at **** from January 2016 to July 2018. Ambulatory LC were compared to well-matched inpatient procedures performed in the same study period. The primary endpoints was the 30-day readmission rate. Secondary endpoints were the discharge rate in the ambulatory group, the post-operative complications rate and cost effectiveness. *Results:* 120/288 (41.7%) patients underwent ambulatory laparoscopic cholecystectomy. Thirty-two (26.7%) patients who underwent ambulatory laparoscopic cholecystectomy had major preoperative comorbidities and 35 (29.2%) had undergone prior abdominal surgery. The readmission rates for ambulatory patients and inpatients were 0.8% and 1.7% (p = 0.56), respectively; 104 (86.7%) ambulatory patients were discharged successfully on the same day. The two groups showed the same post-operative complication rate (p = 0.40). Ambulatory procedures resulted in related cost savings of more than 300% for the hospital and a remarkable financial benefit for the National Italian Healthcare System, accounting for savings exceeding € 27 000 per year. *Conclusions:* Ambulatory laparoscopic cholecystectomy is safe and cost effective. Since a third of ambulatory patients showed comorbidity or previous abdominal surgery, we believe that this procedure may be performed safely in a tertiary HPB centre, even in complex patients.

## Highlights

• Ambulatory laparoscopic cholecistectomy is an efficient, safe and feasible procedure.
• Ambulatory Laparoscopic cholecistectomy may also be offered to complex patients.
• Ambulatory laparoscopic cholecystectomy is cost effective for both Hospital and National Health System.

## 1 Introduction

In recent decades, day case surgery has been performed extensively worldwide. The reason for such popularity is the increasing need to reduce wait times and healthcare costs while guaranteeing patient safety and improving quality of post-operative recovery [1].

Ambulatory surgery has been explored in different abdominal procedures, most of these can ultimately be carried out safety in ambulatory setting [2].

In the United States, almost 90% of hernia repair procedures and 60% of LC are performed in an ambulatory regimen [3], while in the United Kingdom, use of day case surgery regimen has increased in the last years from 67% to 78%, with a significant reduction in overall costs (i.e. average day case cost of £ 698 vs inpatient cost of £ 3375.50) and higher patient satisfaction [4].

Laparoscopic cholecystectomy (LC) is the treatment of choice for symptomatic gallstone disease [5], the most common gastrointestinal disease requiring hospitalization in the United States with an annual estimated cost of almost 5 billion dollars [6]. Ambulatory LC, defined as a same-day admission and patient discharge procedure [7], is widely performed because it is feasible and safe, with a high patient satisfaction rate [8]. Recent data reported a complication and re-admission rate similar to those procedures performed with overnight stays [9], thus providing a cost-effective level of care.

Although the cost effectiveness of day case LC has already been investigated, previous studies were actually weakened by the analysis of old data concerning series mainly performed between 1998 and 2003 [8,[10], [11], [12]], so the current financial benefit of a day case program remains unclear.

The aim of the present study was to evaluate the 30-day readmission rate in ambulatory and inpatient LC. Secondary endpoints were discharge rate within 6 h of surgery of ambulatory LC cases, incidence of post-operative complications and financial benefit of day case vs inpatient LC for the Hospital and the Italian National Healthcare System (NHS).

## 2 Materials and methods

### 2.1 Study design

This is a single-center retrospective cohort study including 288 patients who underwent LC at ****** between January 2016 and July 2018.

Ambulatory LC was defined as a same-day admission and patient's discharge procedure in which surgery was completed by 2 p.m. and hospital discharge performed within 6 h after the end of the operation.

Gallstones disease or gallbladder adenomyomatosis were assessed pre-operatively by ultrasound, CT scan or MRCP. Any non-malignant gallbladder diseases leading to acute/chronic cholecystitis or with history of pancreatitis, common bile duct stones disease and jaundice, dealing with elective LC [identified by International Classification of Diseases (ICD) code 51.23] were included.

The manuscript has been approved from our local Ethical Committee (12/20, February 5, 2020) and registered under researchregistry5358. It has been reported in line with STROCSS criteria [13].

### 2.2 Inclusion criteria

Ambulatory LC was scheduled for patients between 18 and 90 years old with American Society of Anesthesiologists (ASA) physical status classification system [14] grade I-II and body mass index (BMI) <35 kg/m^2^. ASA grade III was considered for ambulatory LC only if patients had well-controlled comorbidities and stable clinical status. History of acute pancreatitis and common bile duct stones disease was not considered as a contraindication.

### 2.3 Exclusion criteria

Patients who did not consent to the ambulatory procedure; patients with ASA grade IV, BMI >35 kg/m^2^ or pre-operative assessment of a high risk of conversion to open technique (i.e. previous supra-mesocolic abdominal surgery) and patients already hospitalized for acute cholecystitis or for another reason were excluded from the study.

### 2.4 Patient matching

Ambulatory LC was compared with well-matched inpatient procedures performed in the same study period. According CRFs analysis, two homogeneous cohorts were identified in order to minimize study bias mainly due to the differences between inpatients and ambulatories. Matching variables included demographics such as age at surgery, gender, BMI (kg/m2), reason for surgery, major comorbidities and ASA score. Finally, of the 167 inpatient procedure, 120 well-matched represented the study control group (i.e. 27 patients were excluded because suffered from more than three major comorbidities, four patients because ASA grade IV, six patients due to BMI≥35 (kg/m2) and 10 patients because age≥85 years old) whereas out of 121 day case LC, one patient only was excluded due to elderly age (namely 88 years old).

### 2.5 Surgery procedure and patient management

Considering both ambulatory and inpatient LC, 182 (75.8%) ones were performed by an HPB consultant surgeon (A.A; L.T; L.T; N.D.L; T.M.M) using a three or four trocars Hasson open technique (one 10-mm trocar in the umbilicus, one 5-mm trocar below the xiphoid process and one or two additional 5-mm ones on the right side). Fifty-eight procedures (24.2%) were performed by mentored surgical trainees.

Routinely outpatient clinic visits were carried out at 1 month (last follow-up) after surgery and blood analyses and ultrasound examinations were performed in order to assess clinical outcomes.

### 2.6 Cost appraisal

#### 2.6.1 Hospital cost

Cost analysis for both ambulatory and inpatient LC was performed by taking into account both fixed costs, such as disposable laparoscopic instrument purchase costs, and variable charges (namely operating theatre and bed day costs). When an overnight stay occurred, the bed day cost was modified according to the length of stay.

Surgical disposable items included 5−10-mm trocars (€ 30.0) and endo-bags (€ 30.0) [Applied Medical®, California, United States], blunt dissectors (€ 11.0) [ConMed®, Utica, United States] and clip appliers (€ 85.0) [Covidien®, Dublin, Ireland]. A standard value-added tax (VAT) of 22% was applied according to the Italian national law.

Procedure time cost was defined as the actual cost per 60 min of operating room usage and consisted of € 240.0/h for an ambulatory theatre and € 600.0/h for an inpatient operating room (the over charge is justified by the difference between the two regimens in number of nurses, operating staff, devices and operating theatre size) [15].

Bedside cost was € 150.0 for an ambulatory patient and € 860.0/day for an inpatient; for those patients who required more than one inpatient day, the daily cost was multiplied by the number of hospitalization days incurred [15].

The overall related cost was thus defined as the sum of previously indicated costs per procedure, multiplied by the number of procedures performed during the study period. Data related to supply costs were collected using acquisition costs provided by the hospital accounting department.

#### 2.6.2 NHS cost

To better address financial burden from an NHS standpoint, a Diagnosis-Related Group (DRG) comparison between day case and inpatient LC was also carried out. According to the current Legislative Decree of October 18, 2012, which established the maximum tariff paid to hospitals with a flat fee (www.gazzettaufficiale.it), the refunded cost of both ambulatory cases and overnight stay LC was € 1458.0. For those LC requiring hospital admission between 1 and 10 days, the DRG refund was € 2834.0 regardless of the length hospitalization stay. Beyond the aforesaid threshold of 10 days, a further daily rate of € 211.0 per patient was then refunded.

### 2.7 Endpoints

Primary outcome was to evaluate the 30-day readmission rate in ambulatory and inpatient LC. Readmission was defined as any hospitalization with a period of at least 24 h occurring within 30 days from the discharge.

Secondary outcomes were discharge rate within 6 h of surgery of the ambulatory LC cases, incidence of post-operative complications and financial benefit of day case vs inpatient LC for the Hospital and the Italian National Healthcare System (NHS). Discharge criteria for ambulatory cases included absence of nausea and vomiting, well-tolerated oral liquids, adequate pain control and ability to pass urine spontaneously. Patients who did not fulfil these criteria were admitted to an inpatient ward for a one-night stay. Complications were recorded according to Clavien-Dindo classification.

### 2.8 Statistical analysis

All data were entered into an Excel database (Microsoft, Redmond, Washington – United States) and the analysis was performed using the Statistical Package for the Social Sciences Windows, version 19.0 (SPSS, Chicago, Illinois, USA). Descriptive statistics consisted of the mean ± standard deviation (SD) for parameters with gaussian distributions (after confirmation with histograms and the Kolgomorov-Smirnov test). Comparison among groups was performed with the ANOVA one-way or the Chi-Square test or Fisher's exact test (if cells<5) for frequencies variables. A p value of <0.05 was considered statistically significant.

## 3 Results

**Fig. 1:**
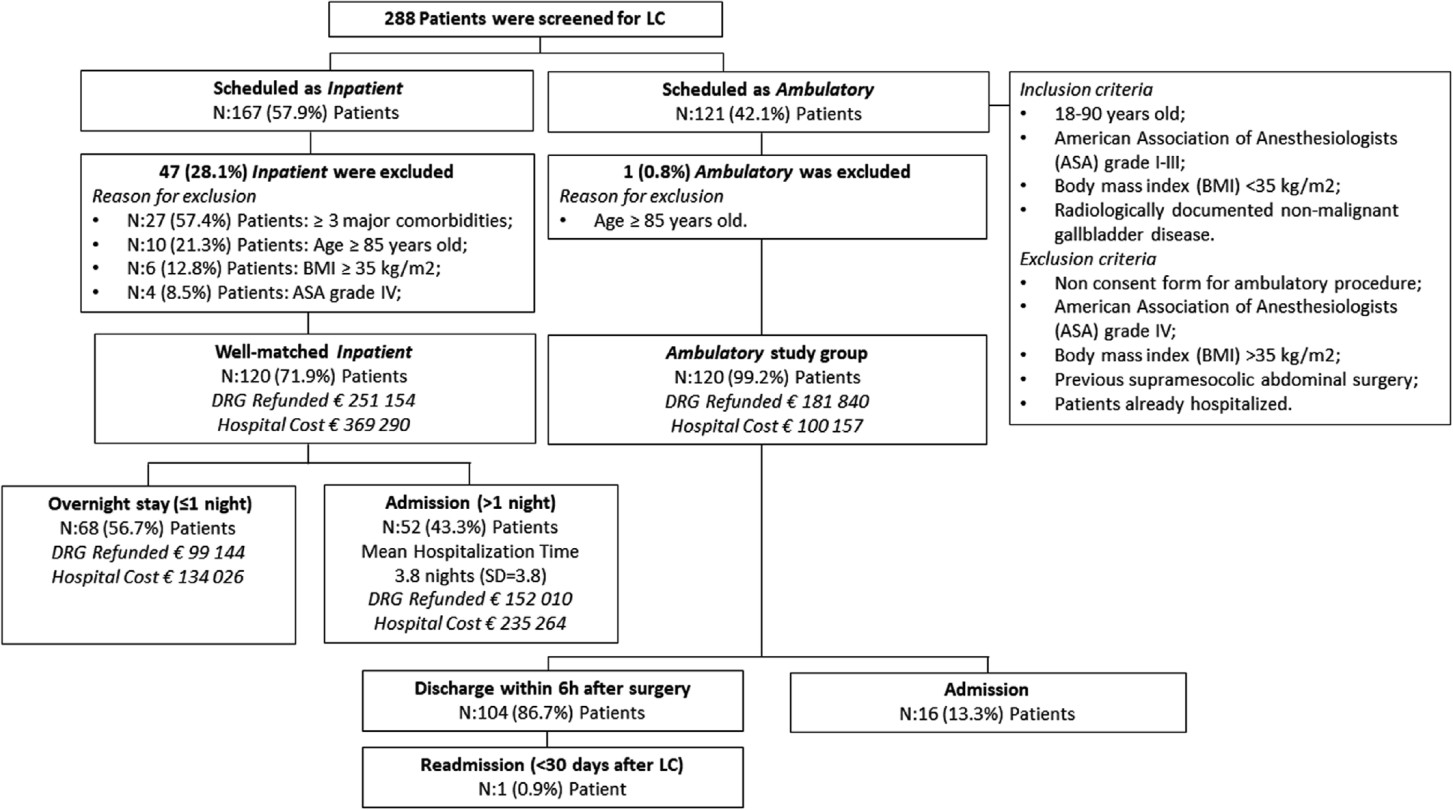
Screening of patients presenting for gallstones disease surgery.

Out of 288 patients, 120 (41.7%) were eligible for ambulatory LC [male = 55 (45.8%), mean age = 52.8 years (SD = 13.1), mean BMI = 26.6 (SD = 4.3) kg/m2]. Of the remaining 167 LC that did not fulfil the previously stated inclusion criteria and thus scheduled for an inpatient procedure, only 120 (71.8%) well-matched patients were considered for the study [male = 64 (53.3%) p = 0.245; mean age = 52.9 (SD = 14.5), p = 0.933; mean BMI = 32.3 kg/m2 (SD = 41.7), p = 0.140] (Fig. 1). Patient demographics, indication for surgery, major comorbidities and ASA scores for both ambulatory and inpatient LC are displayed in Table 1. At referral, 32/120 (26.7%) ambulatory patients had major comorbidities [i.e. cardiovascular diseases (n = 8) (four with permanent atrial fibrillations, three with mild mitral insufficiency and one with moderate tricuspid insufficiency), chronic kidney diseases (n = 5) (three with end-stage kidney diseases and two with “stage 4” chronic kidney disease), respiratory disorders (n = 9) (six with chronic obstructive pulmonary diseases and three with obstructive sleep apnoea) and 10 patients with a non-insulin-dependent diabetes mellitus]. No significant difference was noticed between the two groups according major comorbidities (p > 0.05). Thirty five (29.2%) patients had undergone prior abdominal surgery such as appendectomy (n = 20), open hernia repaired (n = 11) and nephrectomy (n = 2). Two patients had received prior kidney transplantation.

**Table 1:** Patients characteristics of day case vs inpatient LC.

Variables	Day case LC	Inpatient LC	P value
***Number of patients***	*120*	*120*	*-*
***Age (years)***	*52.8 (SD=13.1)*	*52.9 (SD=14.5)*	*0.933*
***Male gender***	*55 (45.8%)*	*64 (53.3%)*	*0.245*
***Body mass index (kg/m***^***2***^***)***	*26.6 (SD=4.3)*	*32.3 (SD=41.7)*	*0.140*
***Indications to surgery***			
*Symptomatic gallstones disease*	*113 (94.2%)*	*112 (93.3%)*	*0.790*
*Gallbladder adenomyomatosis*	*7 (5.8%)*	*8 (6.7%)*	*0.820*
***ASA score***			
*ASA grade 1*	*35 (29.2%)*	*31 (25.8%)*	*0.563*
*ASA grade 2*	*66 (55.0%)*	*55 (45.8%)*	*0.156*
*ASA grade 3*	*19 (15.8%)*	*34 (28.3%)*	*0.020*
***Major comorbidities***			
*Cardiovascular disease*	*8 (6.7%)*	*9 (7.5%)*	*0.801*
*CKD*	*5 (4.2%)*	*8 (6.7%)*	*0.392*
*Respiratory disorders*	*9 (7.5%)*	*6 (5.0%)*	*0.424*
*Diabetes*	*10 (8.3%)*	*8 (6.7%)*	*0.624*
***ERCP pre surgery***			
*14 (11.7%)*			
*25 (20.8%)*			
*0.054*			

Most of ambulatory patients were referred from the surgery outpatient clinic of our Institution (n = 59, 49.2%), the Emergency Department (n = 35, 29.2%) or Gastroenterology Unit (n = 26, 21.6%).

The mean time elapsed from primary referral to surgery for both groups was 2.2 months (SD = 2.8). The main reason for such a long waiting list time was the absence of an LC-dedicated operative theatre in our HPB and transplant unit, resulting in malignant diseases and transplantations taking priority over LC.

Pre-operative ERCP was performed in 14 (11.7%) ambulatory cases and 25 (20.8%) inpatients (p = 0.054).

One hundred one ambulatory patients (84.2%) were ASA grade I or II. Remarkably, 19 (15.8%) ASA III patients were considered for day case LC. The mean operative time in ambulatory vs inpatient LC was 80.9 (SD = 37.8) and 93.2 min (SD = 42.9) (p = 0.450) respectively. The conversion open technique rate in ambulatory and inpatient LC was 3.3% (n = 4) and 8.3% (n = 10) (p = 0.34), respectively.

### 3.1 LC outcome

Among the ambulatory group, one patient (0.8%) was readmitted within 30 days (i.e. 11 post-operative days) due to a gallbladder bed site collection that required antibiotics and percutaneous radiologic drainage. One patient experienced biliary duodenitis, successfully managed by medical therapy in an outpatient clinic setting.

In the inpatient group, two (1.7%) patients were readmitted because of wound dehiscence and gallbladder bed site collection managed by surgical “curettage” and intravenous antibiotics respectively (Table 2). At the one-month scheduled outpatient clinic visit, all patients had good clinical status with normal liver function tests and ultrasound evaluation. Two patients experienced a trocar site incisional hernia within 12 months after surgery.

**Table 2:** Readmission rate and post operative complications of day case vs inpatient LC.

Variables	Day case LC	Inpatient LC	P value
***Number of patients***	*120*	*120*	*-*
***Readmission event (within 30 days)***	*1 (0.8%)*	*2 (1.7%)*	*0.561*
***Complications***	*2 (1.7%)*	*4 (3.3%)*	*0.408*
*Clavien Dindo Grade 1*	*1 (0.8%)*	*0*	*0.316*
*Clavien Dindo Grade 2*	*0*	*1 (0.8%)*	*0.316*
*Clavien Dindo Grade 3*	*1 (0.8%)*	*3 (2.5%)*	*0.313*

One hundred and four (86.7%) ambulatory patients were successfully discharged within 6 h after surgery while 16 (13.3%) patients required overnight admission. The reasons for overnight admission were conversion to laparotomy (n = 4), post-operative pain (n = 4), drain output (n = 2), fever (n = 1) or patient's request (n = 5).

### 3.2 Cost evaluation

#### 3.2.1 Hospital cost

Ambulatory LC resulted in a total hospital expenses of € 100 156.8 considering both admission and readmission events [mean tariff paid per patient: € 833.7 (SD = 465.9)] *vs* € 369 290.0 [mean tariff paid per patient: € 3077.4 (SD = 2740.1)] in inpatient surgery (p = 0.0001).

Disposable laparoscopic instrument purchase costs accounted for € 263.50 of included VAT for the three trocars LC technique. If an additional port was necessary, instrument-purchase fixed costs were then adjusted to € 300.10. This resulted in almost € 34 696.8 for ambulatory LC [(€ 263.5 × 35 three-trocar LC performed) + (€ 300.1 × 85 four-trocar LC performed)] vs € 34 770.0 for the inpatient approach [(€ 263.5 × 34 three-trocar LC performed) + (€ 300.1 × 86 four-trocar LC performed)].

Operative time cost was € 38 860.0 for day cases vs € 111 780.0 for inpatient LC. Considering length of stay, the overall cost of ambulatory cases was € 26 600.0 vs € 222 740.0 for inpatients [mean inpatient length of stay: 2 (SD = 3.0) days].

#### 3.2.2 NHS cost

The overall NHS costs for ambulatory and inpatients cases were € 181 840.0 and 251 154.0, respectively (p = 0.0001). The mean ambulatory LC DRG was € 1515.3 (SD = 276.1) vs € 2092.9 (SD = 798.4) per inpatient, thus resulting in NHS mean over-charge (namely Δ DRG) of € 577.6 per inpatient procedure (Δ DRG = € 2092.9 - € 1515.3 respectively). This translated to a total overcharge for the Italian NHS of € 69 312.0 (€ 577.6 × 120 LC) when ambulatory regimen was not considered (Table 3).

**Table 3:** Composite cost evaluation.

Variables	Day case LC	Inpatient LC	P value
***Number of patients***	***120***	***120***	***-***
***Hospital Cost per patient***	***€ 833.7 (SD = 465.9)***	***€ 3077.4 (SD = 2740.1)***	***0.0001***
*Instruments purchase cost*	*€ 288.2 (SD= 17.2)*	*€ 289.8 (SD= 16.6)*	*0.485*
*Operative time cost*	*€ 323.8 (SD= 151.3)*	*€ 931.50 (SD= 429.72)*	*0.0001*
*Bed side cost*	*€ 221.7 (SD= 362.7)*	*€ 1856.2 (SD= 2549.7)*	*0.0001*
***DRG reimbursement per patient***	***€ 1515.3 (SD = 276.1)***	***€ 2092.9 (SD = 798.4)***	***0.0001***
***Total Hospital Cost***	***€ 100 156.8***	***€ 369 290.0***	***0.0001***
***Total DRG Reimbursement***	***€ 181 840.0***	***€ 251 154.0***	***0.0001***

## 4 Discussion

LC is considered an efficient, safe and low-morbidity procedure [[16], [17], [18]]. Ambulatory LC allows short post-operative hospitalization, possibility of early return to the daily activities with a positive impact on patient's quality of life [19]. It also contributes to a reduction in the waiting list time and an increase in the number of inpatient beds that could be used for additional services, thus providing a better utilization of limited healthcare resources. Patients who underwent ambulatory LC showed a high satisfaction, low unplanned admission and similar readmission rates vs those who underwent standard inpatients LC. Surprisingly, although some authors reported a cost effectiveness between 11% and 46% when compared with inpatients procedures [10,18,20,21], this did not ultimately result in significantly cost savings [8]. Recently, Vaughan J et al. showed no difference in terms of adverse events (RR 3.24; 95% CI 0.74 to 14.09), quality of life (SMD -0.11; 95% CI -0.33 to 0.10), pain control (MD 0.02 cm visual analogue scale score; 95% CI -0.69 to 0.73), time to return to activity (MD -0.55 days; 95% CI -2.18 to 1.08) or work (MD -2.00 days; 95% CI -10.34 to 6.34) as well as hospital readmission rate (RR 1.09; 95% CI 0.33 to 3.60) and failed discharge (RR 0.96; 95% CI 0.65 to 1.41) between day case LC and overnight stay [22]; therefore the ambulatory procedure seems to be as safe as an overnight approach, but without improvement in clinical outcomes.

A careful selection of patients is considered mandatory in order to obtain a high success rate and a low complication rate [23,24]. Several risk factors such as age>50 years, ASA grade III, longer operative time, previous abdominal surgery, history of acute pancreatitis and cholecystitis have been identified as predictors of day case failure [21,25]. In our series more than 25% of patients presented high comorbidities at surgery, such as cardiovascular and pulmonary disorders (n = 17) or chronic kidney disease in haemodialysis treatment (n = 5) or were classified as ASA III (n = 19). Since the successful same-day discharge rate reached almost 70% in this subgroup, we can assert that ambulatory LC in a Tertiary Centre may also be offered also to complex patients, usually considered too high risk. In patients without comorbidity or gallstones disease complications the ambulatory LC program could be also applied in Hospital with lower LC surgery activity.

A recent systematic review on more than 600 day cases LC showed an inpatients and readmission rates of 13% and 2.4% respectively and a post-operative complication rate of about 5% [8]. Our admission rate was in accordance with those findings, since 13% of patients required hospitalization after an ambulatory procedure; however we observed a lower readmission rate (0.8%).

To the best of our knowledge this is the first report to address the financial burden of ambulatory LC on both Hospital and NHS sides in Italy. The hospital costs were calculated for ambulatory and inpatient LC and resulted in expenses of € 100 156.8 and € 369 290.0, respectively. Furthermore, the inpatient LC represents a major expense for NHS because in the Lazio Region (Italy), the mean reimbursement for ambulatory and hospitalized LC reaches € 1515.3 vs € 2092.9, respectively, resulting in NHS mean over-charge of € 577.6 for each inpatient LC. Finally, we can assert that ambulatory LC represented for our Hospital from January 2016 to July 2018 a profit of € 81 683.2 and a loss making of € 118 136.0 when inpatient LC were considered (total DRG reimbursement - Hospital cost). The Hospital paramedical and medical cost savings were described by Topal B et al., in 98 patients without systemic disease that did not required supplementary post-operative care [11]. In this cohort of patients, a final cost effectiveness of more than € 50 000 was reported, taking into account the time of each medics and paramedics spent in the Activity Centre and the pharmaceutical costs. This resulted in a cost effectiveness of approximately 40% compared with inpatients LC.

Considering the social magnitude of gallstones disease, financial implications for healthcare services should always be considered. We report herein the results of a composite cost effectiveness evaluation obtained considering surgery-related costs including disposable sets, operative and bed side costs as well as DGR refunded fees and thus provides a full financial assessment that takes into consideration both local tertiary care centre and NHS.

Since the ambulatory LC has been the standard of care in the United States for over 20 years, we are aware that the current study doesn't add novelty regarding the feasibility and safety of ambulatory LC; however we are also convinced on the importance of proving its safety and saving impact -updated to the recent years - in different public health care systems, such as in Italy. The retrospective analysis and the different patients characteristics between the groups represents a further limitation of our study. We attempted to manage this drawback considering, as stated above, only inpatient LC matched with ambulatory cases that permitted us to overcome this drawback.

Finally, we could assume an hospital cost savings of about € 170.000 if inpatient LC (n:120) had been performed in an ambulatory setting.

## 5 Conclusion

The 2-year ambulatory LC experience at ****** finally resulted in more than 300% monetary savings and about 70% of cost effectiveness for the NHS, namely € 27 724.8 cost savings per year. These significant clinical and financial benefits must always be considered in order to promote, in well-selected centres, a day case program, even in the high-morbidity patients.

## Ethical approval

The manuscript has been approved from the Ethical Committee of Fondazione PTV, Policlinico Tor Vergata, Rome (12/20, February 5, 2020).

## Sources of funding

This research did not receive any specific grant from funding agencies in the public, commercial, or not-for-profit sectors.

## Author contribution

Giovanni Monteleone and Giuseppe Tisone were responsible for the conception, design and analysis of the study and for editing the final report; Tommaso Maria Manzia and Claudia Quaranta wrote the paper; Vincenzino Filingeri, Luca Toti, Alessandro Anselmo, Laura Tariciotti, Gerardo De Carolis, Roberto Sorge, Nicola Di Lorenzo and Roberta Angelico were involved with the collection and interpretation of data.

## Research registration Unique Identifying number (UIN)

This study was registered under researchregistry.com. Unique Identifying number (UIN): researchregistry5358. https://www.researchregistry.com/browse-the-registry#home/registrationdetails/5e44310aac8e6100156025bd/

## Guarantor

Giuseppe Tisone.

## Provenance and peer review

Not commissioned, externally peer reviewed.

## Declaration of competing interest

None of the authors has to declare conflict of interest in relation with the presented data in this paper.
